# Supplementary material for: Six states of Enterococcus hirae V-type ATPase reveals non-uniform rotor rotation during turnover
Source: Commun Biol. 2023 Jul 28;6:755. doi: 10.1038/s42003-023-05110-8 (PMC10382590; doi:10.1038/s42003-023-05110-8)
Supplement: Supplementary file 2 — Supplementary Information [file 42003_2023_5110_MOESM2_ESM.pdf]

Six states of *Enterococcus hirae* V-type ATPase reveals non-uniform rotor rotation during turnover

Burton-Smith et al.

Additional Information

Following Supplementary Material is available for this paper.

- Supplementary Figures 1-12 (*available in this file*)
- Supplementary Tables 1 (*available in this file*)

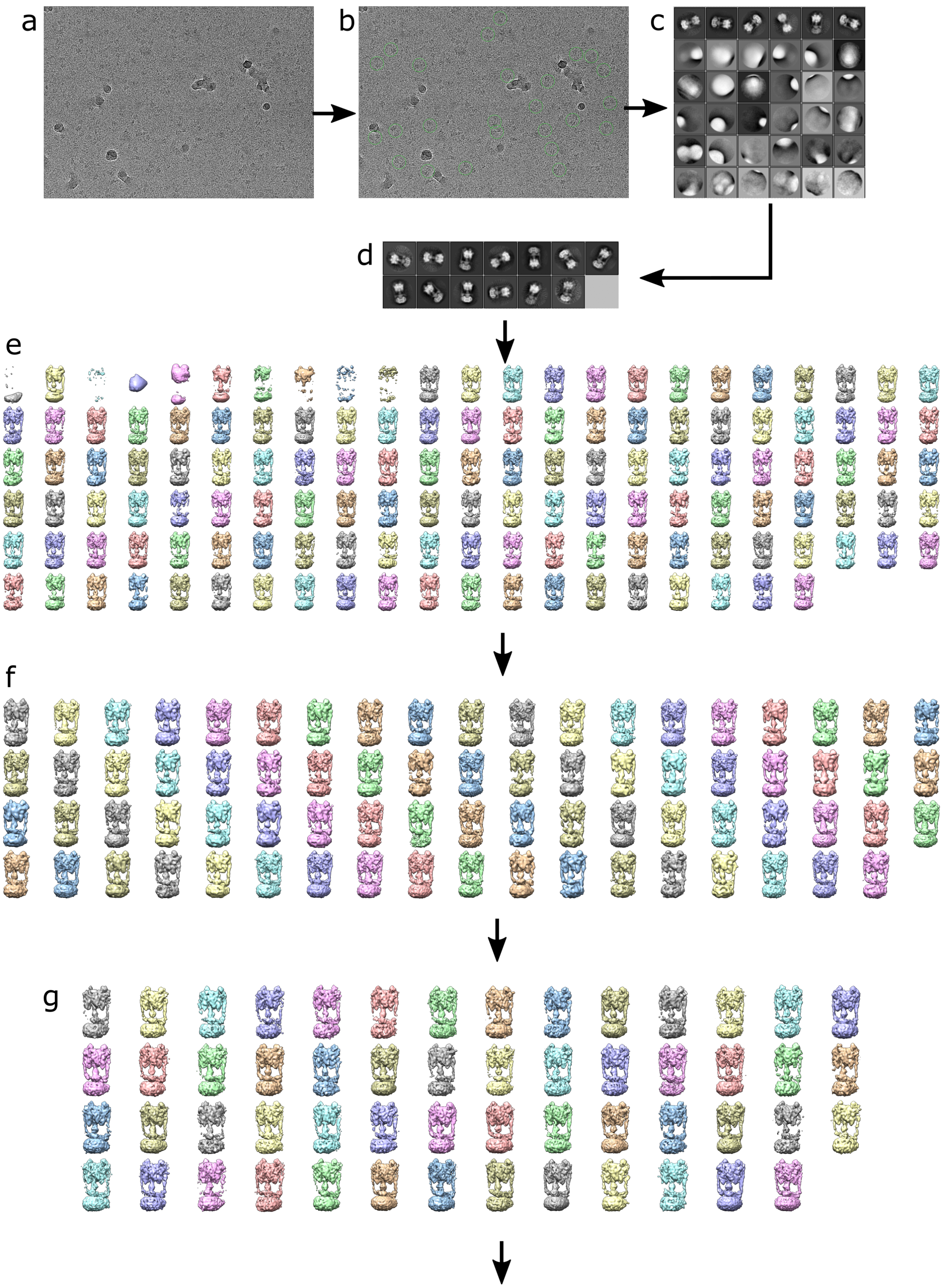
a
b
c
d
e
f
g

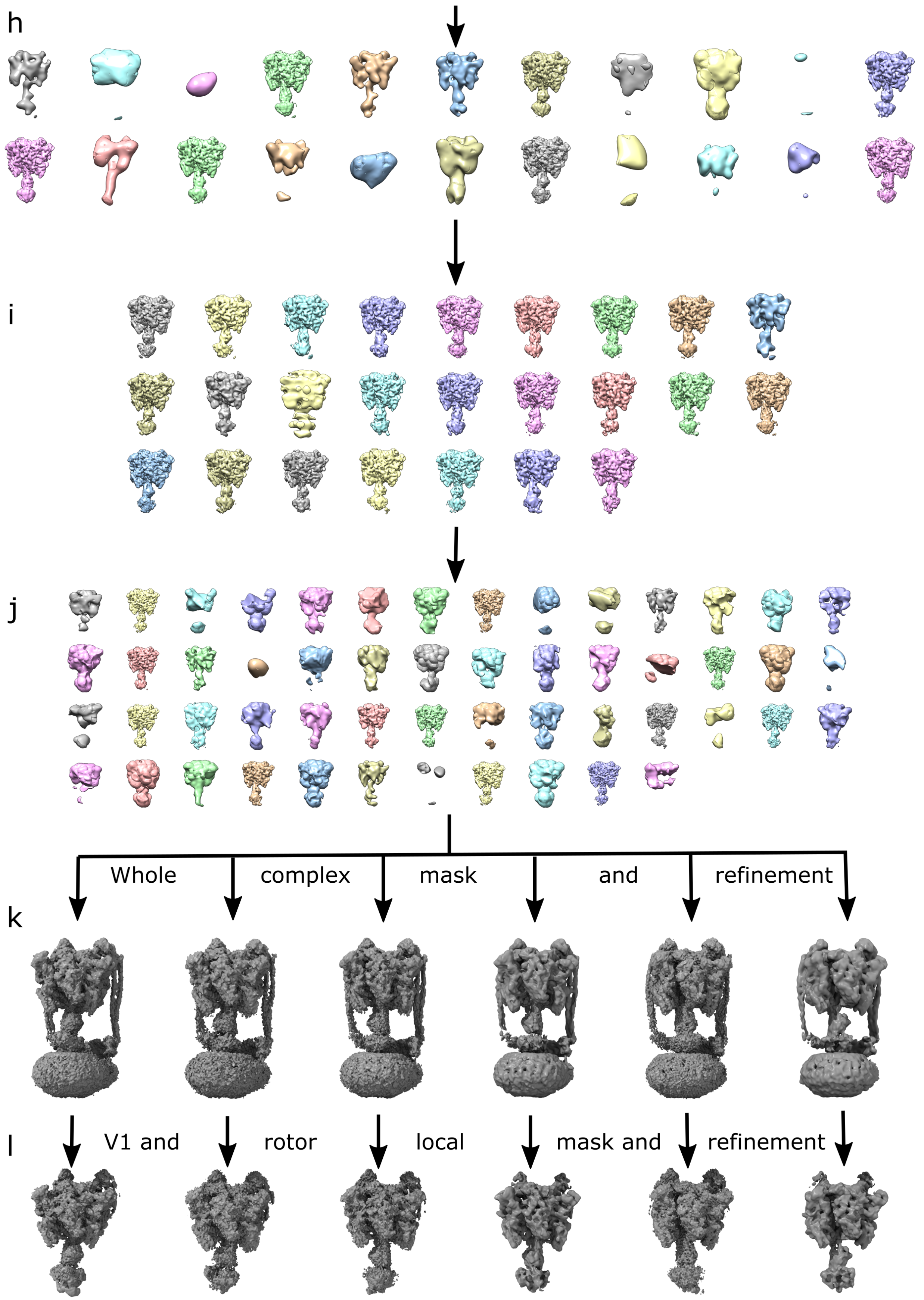
h
i
j
Whole complex mask and refinement
k
l
V1 and rotor local mask and refinement

Supplementary Fig. 1. Flowchart showing the processing pathway. a) A representative micrograph. b) The same micrograph with autopicked particles circled in green. c) Classes from a 2D classification of a subset of the picks. d) After recombining all selected 2D classes from the subset runs, a second round of 2D classification was carried out and all classes recognisable as V-ATPase were selected. e) All 3D classes from individual rounds of 3D classification; all classes with a complete V_o domain and rotor were selected. f) All unbroken classes were combined into a single 3D refinement, which was then classified with alignment disabled, classes with features missing were discarded. g) Further 3D classification with alignment disabled. h) A soft mask on the V_1 domain was created, and 3D classification carried out, with high-occupancy and high-resolution classes selected. i) After grouping by F-subunit orientation, further 3D classification with alignment disabled was carried out. j) Further selection by F-subunit orientation of classes. 3D classification with alignment disabled was used with each independent orientation of the F-subunit (all classes shown) and was used for a final check. A few classes contained just one or two particles were discarded. k) After generation of a reference with "relion_reconstruct" for each orientation, the complex of each state was passed to 3D refinement and underwent cycles of 3D refinement and CTF refinement until no further resolution improvement was evident. l) Using the V_1-focussed mask, V_1 domain refinements were carried out on the final particle sets for each orientation. FSC curves of the respective reconstructions are shown in Fig. S3.

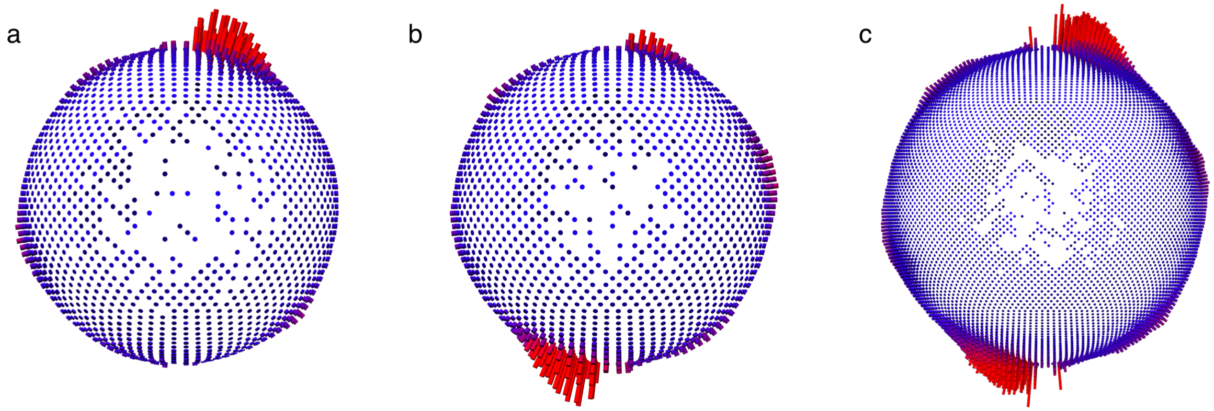

Supplementary Fig. 2. Angular assignments showing the orientation preference for the detergent solubilised EhV-ATPase. a) Angles for a 3D refinement from the first dataset. b) Angles from the second and third datasets. c) Combining all datasets permitted the refinements to converge at a finer angular sampling.

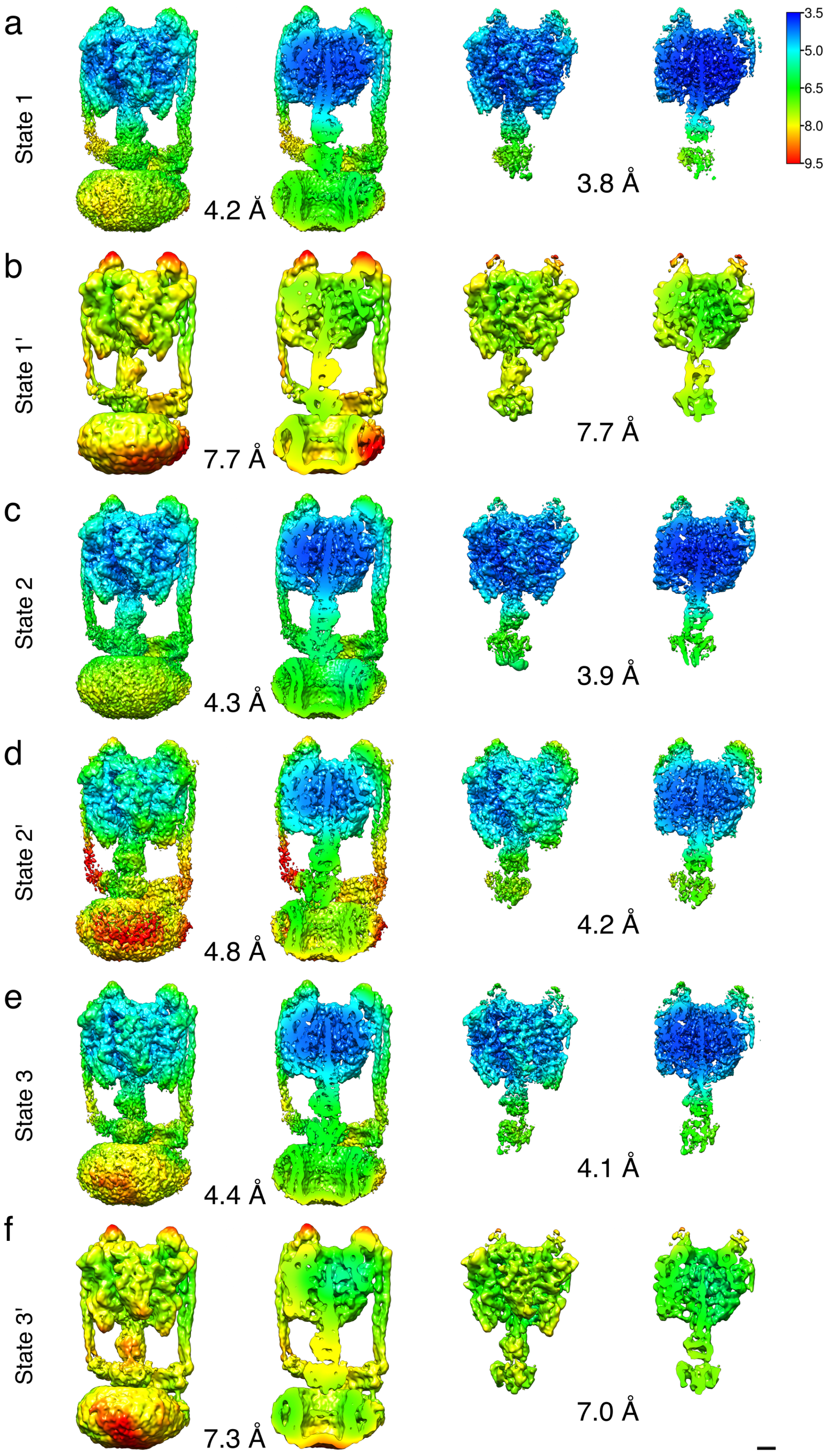
a
State 1
4.2 Ǎ
3.8 Å
3.5
5.0
6.5
8.0
9.5
b
State 1'
7.7 Å
7.7 Å
c
State 2
4.3 Å
3.9 Å
d
State 2'
4.8 Å
4.2 Å
e
State 3
4.4 Å
4.1 Å
f
State 3'
7.3 Å
7.0 Å

Supplementary Fig. 3. Reconstructions of the six states of EhV-ATPase. The maps are filtered and coloured by local resolution as calculated with the local resolution function of RELION 3.1. a) State 1 showing the whole map, the whole map sliced vertically to allow visualisation of internal density and resolution, the V_1 focussed refinement, and the V_1 focussed refinement sliced vertically to allow visualisation of internal density and resolution. The maps are displayed in the same way as State 1 (a) for State 1' (b), Stare 2 (c), State 2' (d), State 3 (e), and State 3' (f), respectively. Scale bar equals 2 nm.

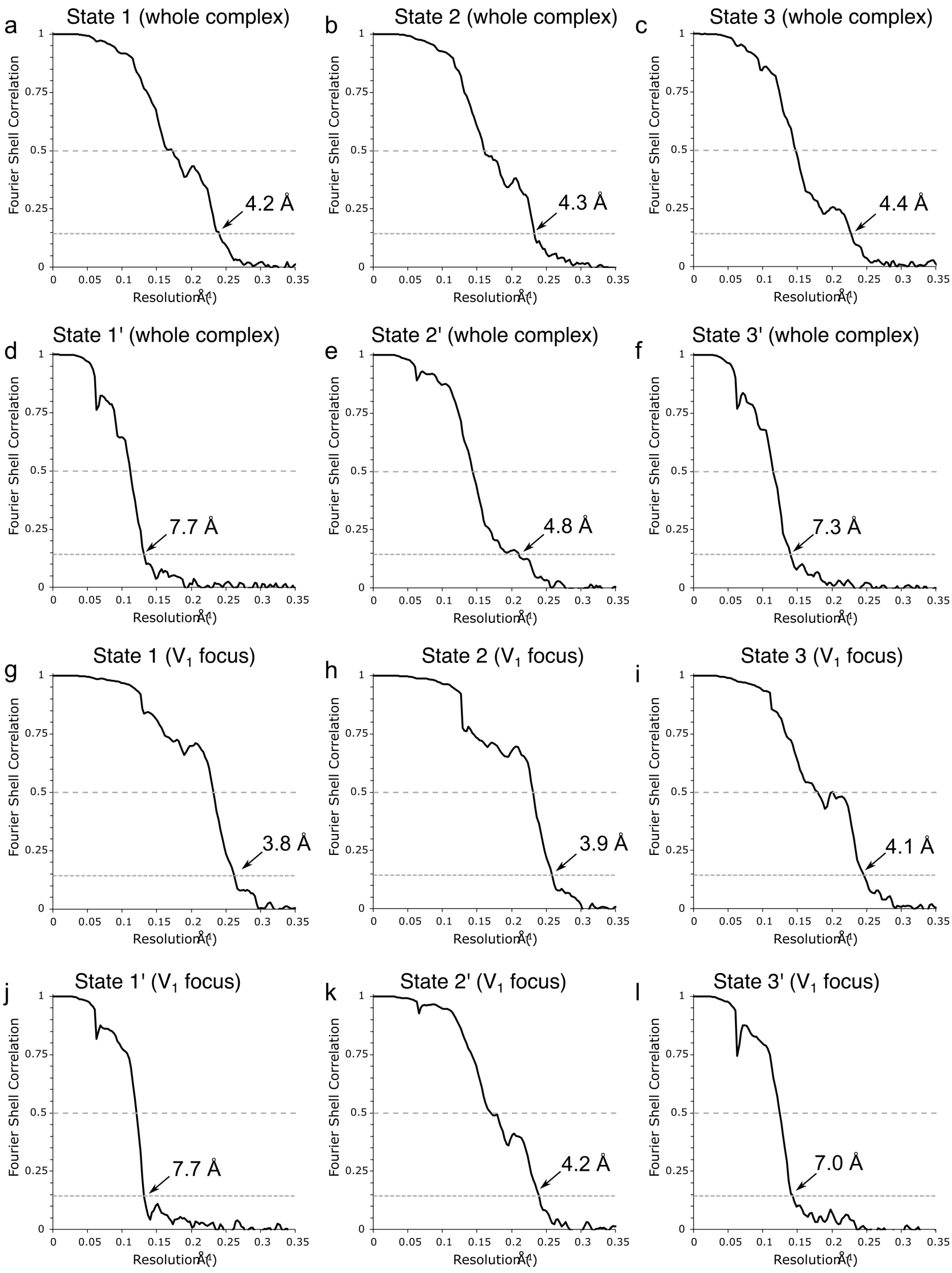
a
State 1 (whole complex)
4.2 Å
b
State 2 (whole complex)
4.3 Å
c
State 3 (whole complex)
4.4 Å
d
State 1' (whole complex)
7.7 Å
e
State 2' (whole complex)
4.8 Å
f
State 3' (whole complex)
7.3 Å
g
State 1 (V_1 focus)
3.8 Å
h
State 2 (V_1 focus)
3.9 Å
i
State 3 (V_1 focus)
4.1 Å
j
State 1' (V_1 focus)
7.7 Å
k
State 2' (V_1 focus)
4.2 Å
l
State 3' (V_1 focus)
7.0 Å
Fourier Shell Correlation
Resolution (Å^{-1})
0
0.05
0.1
0.15
0.2
0.25
0.3
0.35
0.25
0.5
0.75
1

Supplementary Fig. 4. Fourier Shell Correlation curves of the twelve reconstructions. a) Whole complex EhV-ATPase at State 1 at 4.2 Å. b) Whole complex EhV-ATPase at State 2 at 4.3 Å. c) Whole complex EhV-ATPase at State 3 at 4.4 Å. d) Whole complex EhV-ATPase at State 1' at 7.7 Å. e) Whole complex EhV-ATPase at State 2' at 4.8 Å. f) Whole complex EhV-ATPase at State 3' at 7.3 Å. g) V_1 domain EhV-ATPase at State 1 at 3.8 Å. h) V_1 domain EhV-ATPase at State 2 at 3.9 Å. i) V_1 domain EhV-ATPase at State 3 at 4.1 Å. j) V_1 domain EhV-ATPase at State 1' at 7.7 Å. k) V_1 domain EhV-ATPase at State 2' at 4.2 Å. l) V_1 domain EhV-ATPase at State 3' at 7.0 Å. All resolutions reported at FSC=0.143.

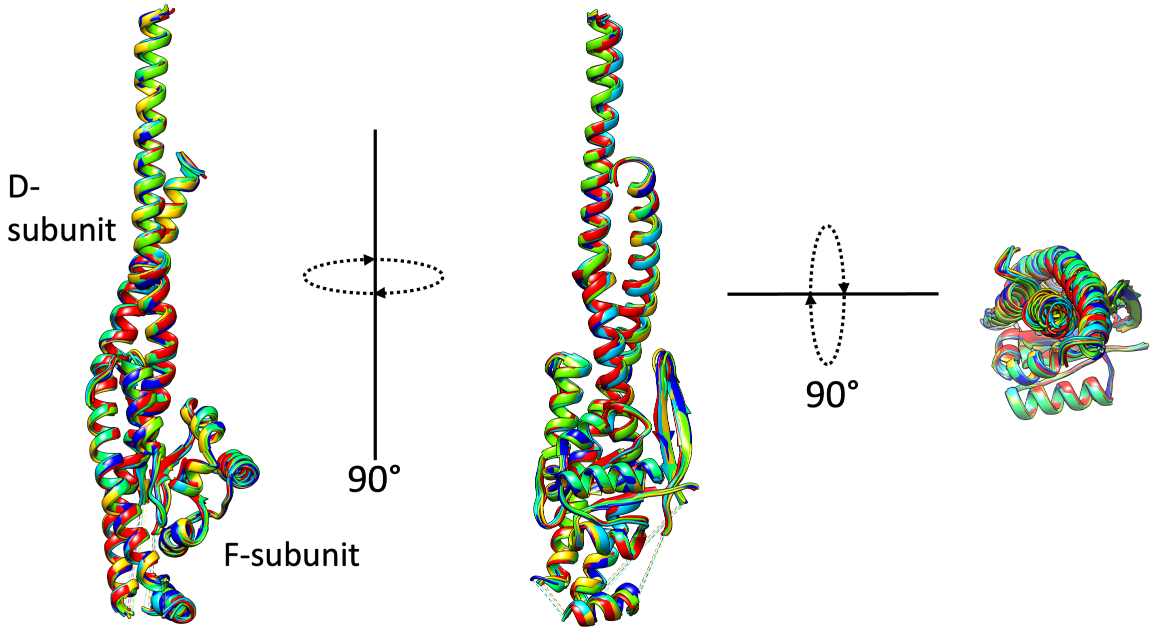

Supplementary Fig. 5. The molecular models of the rotor D-subunits and the F-subunits. The models fitted into the cryo-EM maps of the six states were overlayed. The structural models of the D- and F-subunits were derived from the crystal structure of the 3 ADP-bound V_1 complex (PDBID: 5KNC), and fitted into the cryo-EM maps of each state after modification.

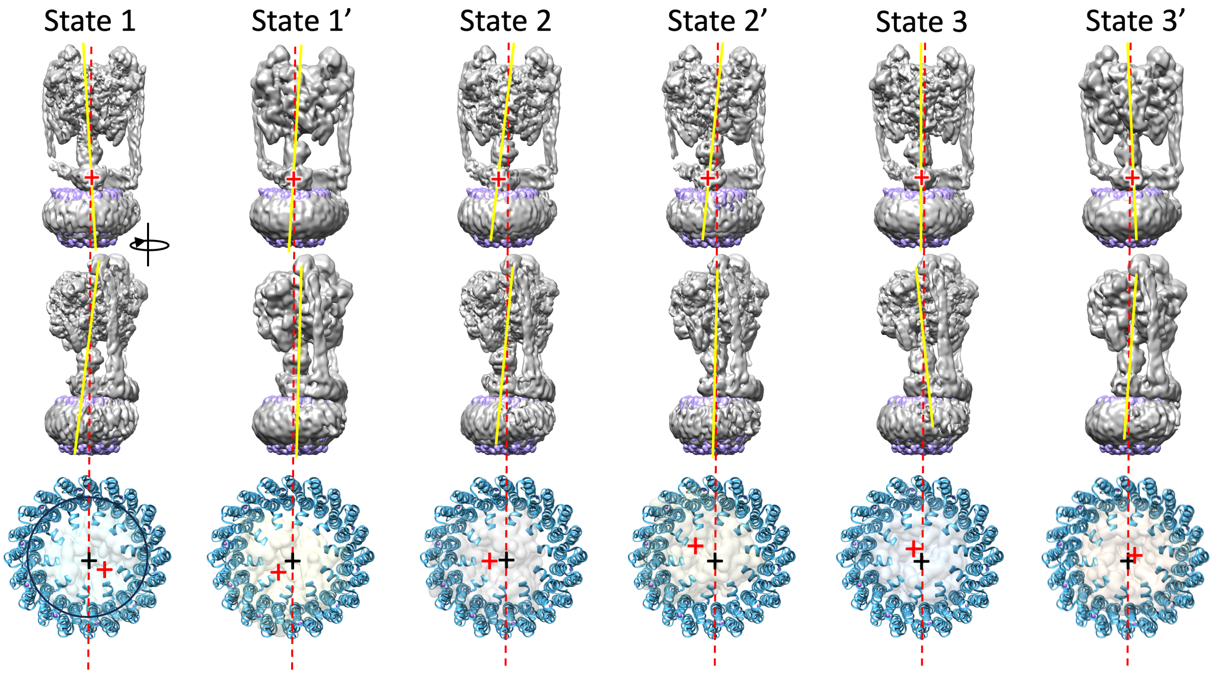

Supplementary Fig. 6. The off-axis rotation of the rotor in six states. Each state map of EhV-ATPase is aligned to the c-ring in UCSF Chimera via the "molmap" command and "fit in map" function applied to PDBID:2BL2. The dashed red lines indicate the vertical centre axis of the c-ring. A solid yellow line is drawn along the axis of the rotor. All six states are viewed from parallel to the membrane (a-subunit furthest away from viewer) (top row) and rotated 90° (middle row). Bottom row; PDB model 2BL2 (c-ring) using "molmap" to generate the densities of the d-subunit, showing their position relative to the centre of the c-ring. The centre of the c-ring on each state is marked with a black cross in bottom row, while the volume centre on the d-subunit is marked with a red cross in top and bottom rows.

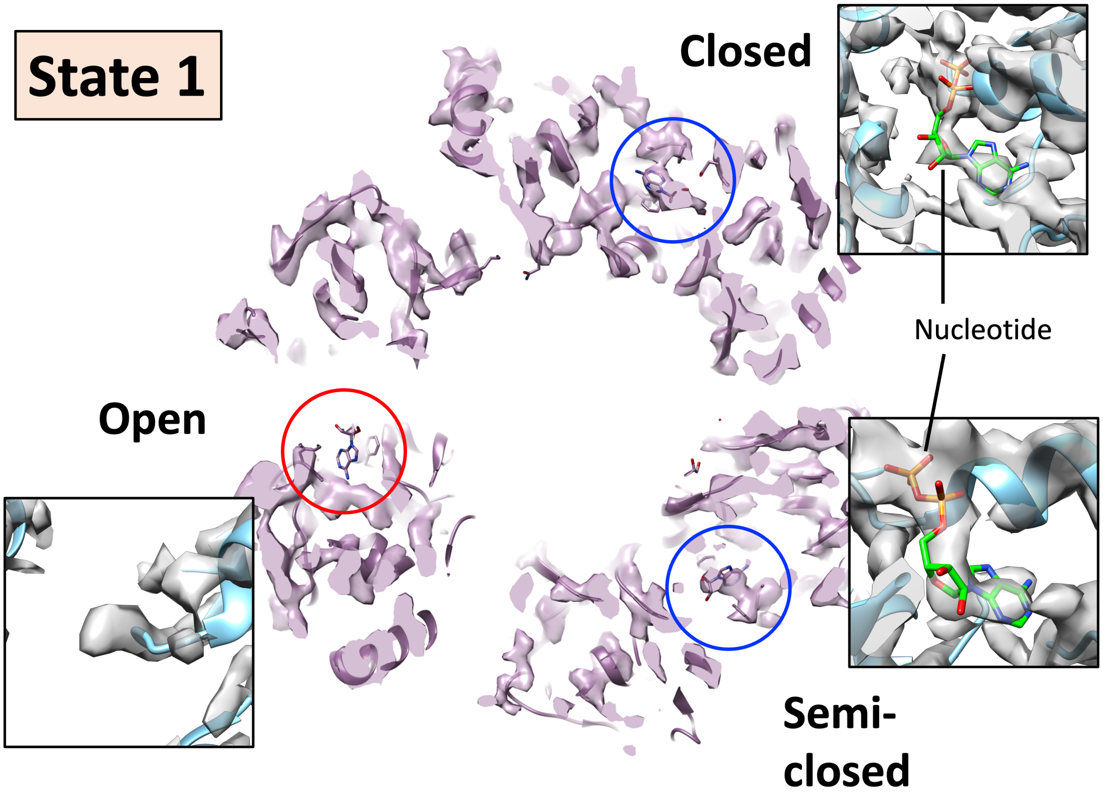

Supplementary Fig. 7. The nucleotide binding pocket densities in EhV-ATPase State 1. The figure is magnified from Fig. 6. The map is sliced through the density map at the level of the nucleotide binding pocket, and with PDBID:5KNC fitted to check whether density for a bound nucleotide is present or not. Blue circles indicate the presence of density corresponding to the bound nucleotide in the fitted PDB, red circles indicate missing density (only nucleotide from PDB is visible). Each nucleotide binding pocket is magnified as inset.

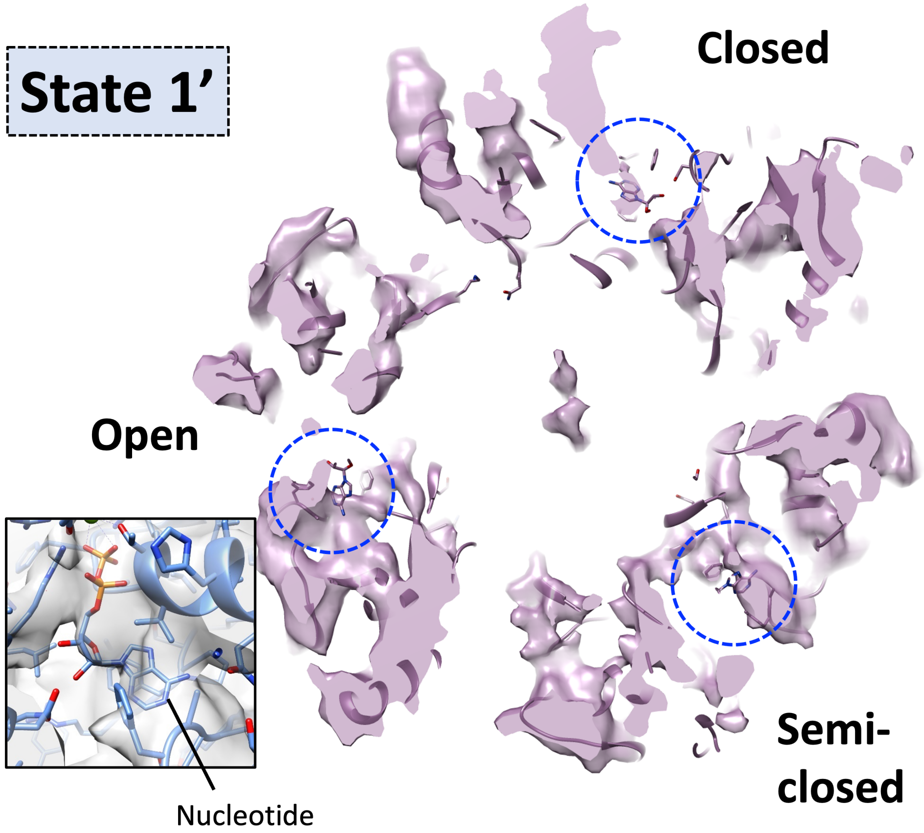

Supplementary Fig. 8. The nucleotide binding pocket densities in EhV-ATPase State 1’.

The figure is magnified from Fig. 6. The map is sliced through the density map at the level of the nucleotide binding pocket, and with PDBID:5KNC fitted to check whether density for a bound nucleotide is present or not. Blue circles indicate the presence of density corresponding to the bound nucleotide in the fitted PDB. The nucleotide binding pocket of the “Open” conformation is further enlarged and the map is displayed at 9 σ density, as inset. It shows difficulty in clear determination of binding geometry in binding pockets for low resolution states (1’ and 3’).

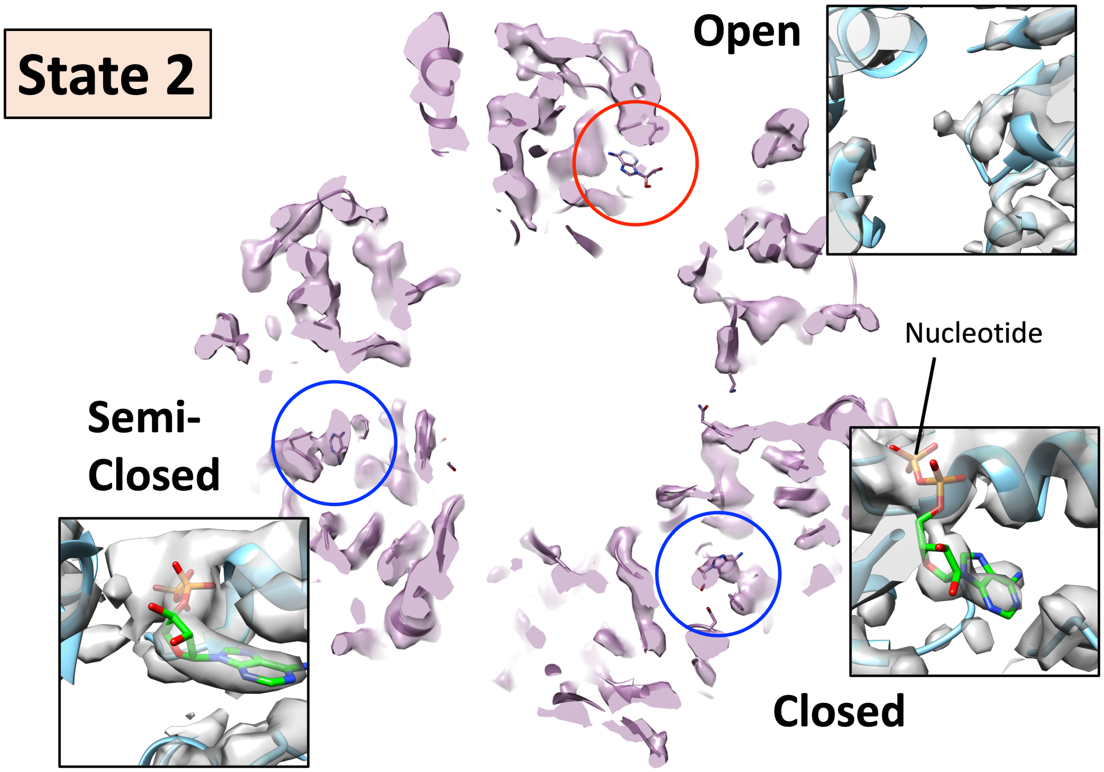

Supplementary Fig. 9. The nucleotide binding pocket densities in EhV-ATPase State 2. The figure is magnified from Fig. 6. The map is sliced through the density map at the level of the nucleotide binding pocket, and with PDBID:5KNC fitted to check whether density for a bound nucleotide is present or not. Blue circles indicate the presence of density corresponding to the bound nucleotide in the fitted PDB, red circles indicate missing density (only nucleotide from PDB is visible). Each nucleotide binding pocket is magnified as inset.

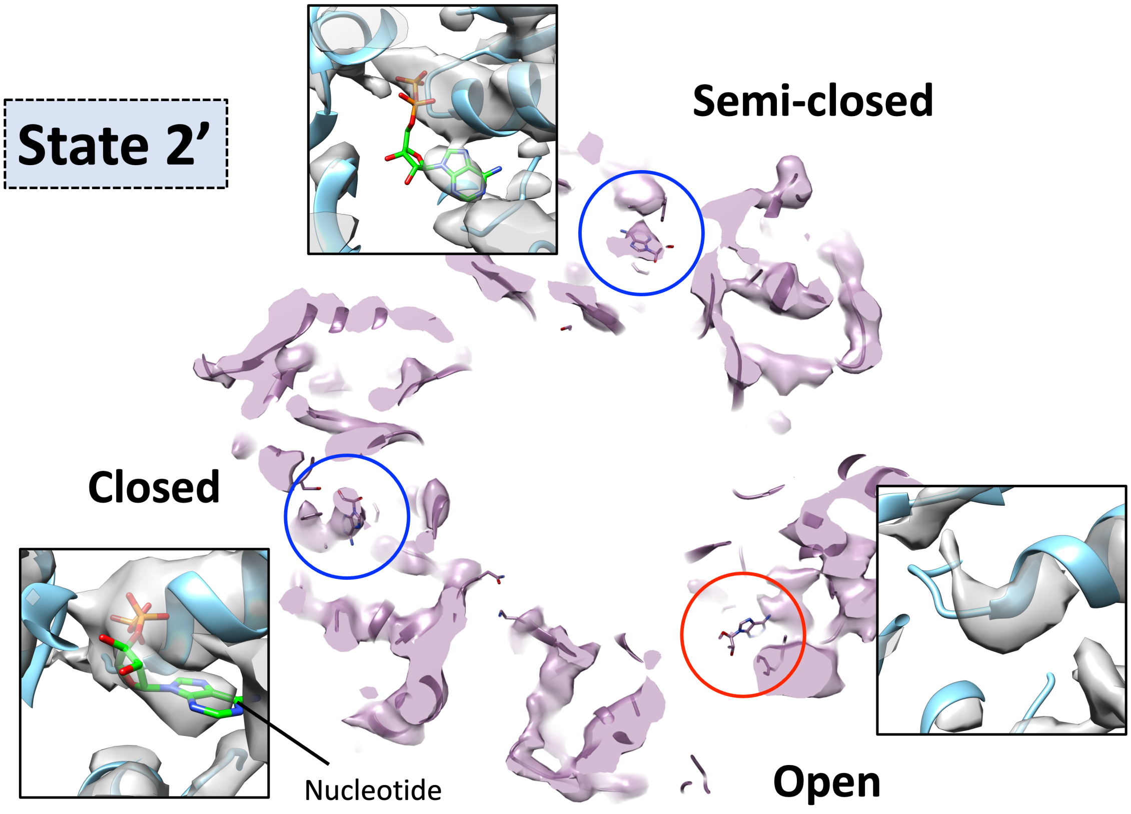

Supplementary Fig. S10. The nucleotide binding pocket densities in EhV-ATPase State 2'. The figure is magnified from Fig. 6. The map is sliced through the density map at the level of the nucleotide binding pocket, and with PDBID:5KNC fitted to check whether density for a bound nucleotide is present or not. Blue circles indicate the presence of density corresponding to the bound nucleotide in the fitted PDB, red circles indicate missing density (only nucleotide from PDB is visible). Each nucleotide binding pocket is magnified as inset.

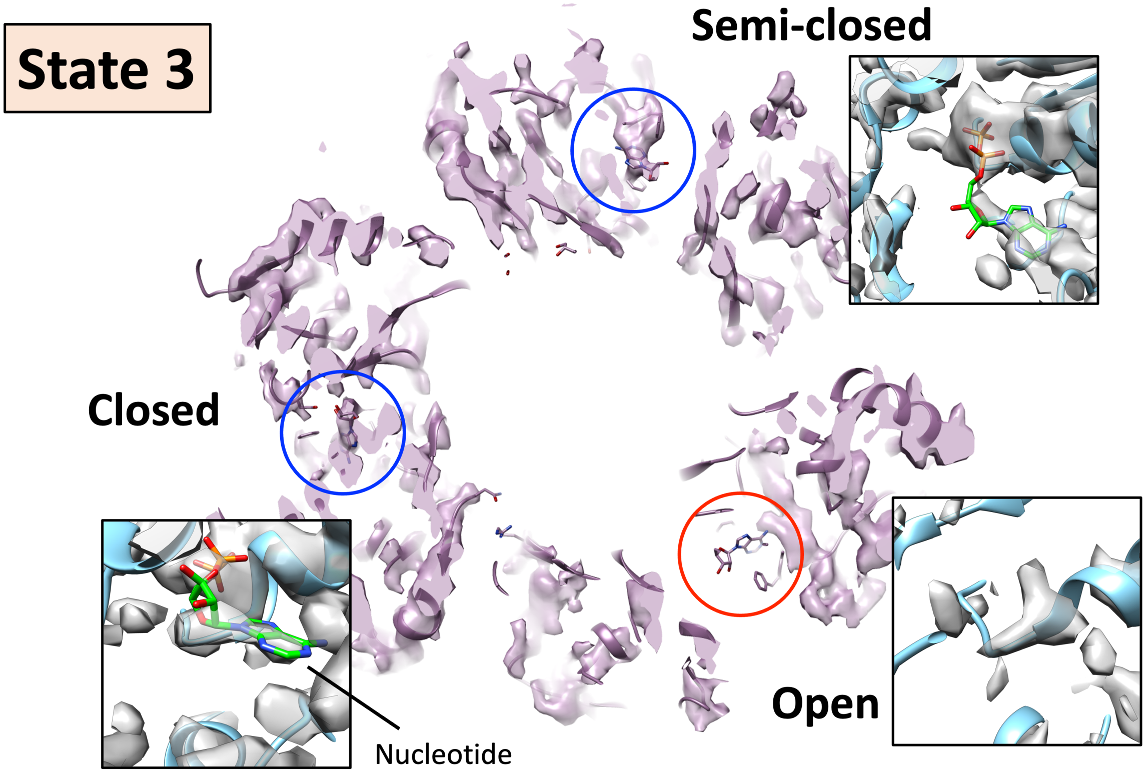

Supplementary Fig. 11. The nucleotide binding pocket densities in EhV-ATPase State 3. The figure is magnified from Fig. 6. The map is sliced through the density map at the level of the nucleotide binding pocket, and with PDBID:5KNC fitted to check whether density for a bound nucleotide is present or not. Blue circles indicate the presence of density corresponding to the bound nucleotide in the fitted PDB, red circles indicate missing density (only nucleotide from PDB is visible). Each nucleotide binding pocket is magnified as inset.

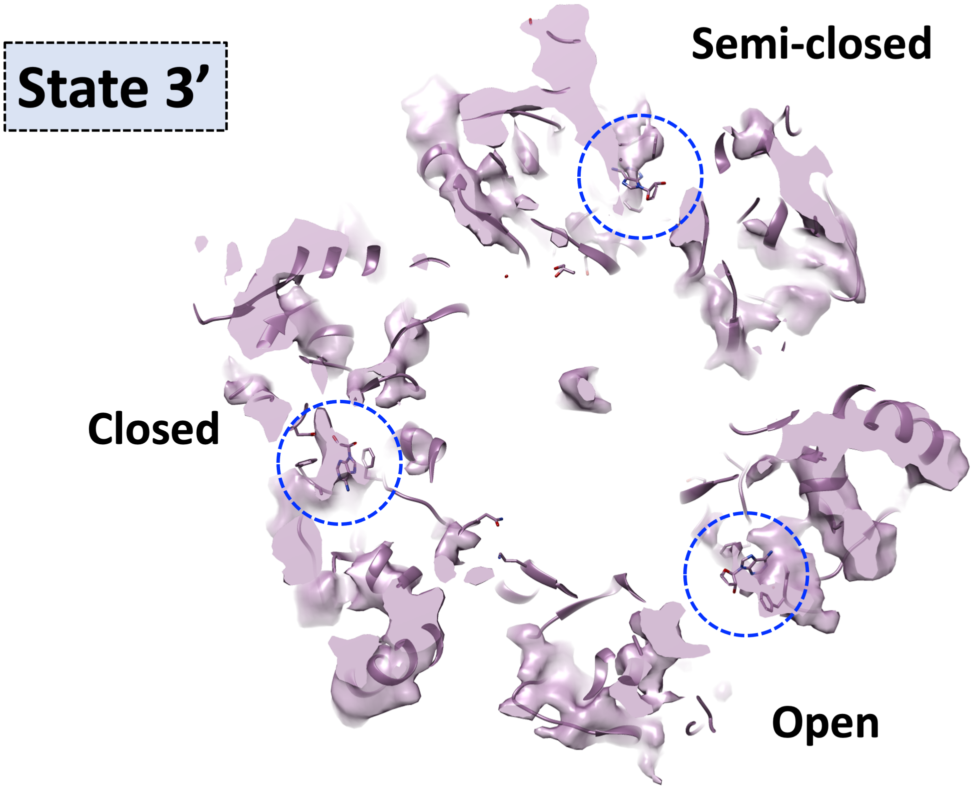

Supplementary Fig. 12. The nucleotide binding pocket densities in EhV-ATPase State 3'. The figure is magnified from Fig. 6. The map is sliced through the density map at the level of the nucleotide binding pocket, and with PDBID:5KNC fitted to check whether density for a bound nucleotide is present or not. Blue circles indicate the presence of density corresponding to the bound nucleotide in the fitted PDB. As shown in Fig. S8 inset, it is difficult in clear determination of binding geometry in binding pockets for low resolution states (1' and 3').

Dataset	Set 1	Set 2	Set 3
**Name**	EhV-ATPase with ATP, Mg^{2+}, and Na^{+}	EhV-ATPase with ATP, Mg^{2+}, and Na^{+}	EhV-ATPase with ATP, Mg^{2+}, and Na^{+} (tilt acquired)
**Microscope**	JEOL CRYO ARM 300		
**Detector**	Gatan K3 (5760 × 4092)		
**Magnification**	50,000× (equivalent to 1.01 Å/pixel)		
**Target Defocus**	-1 ~ -2 μm		
**Electron dose**	60	60	50
**Total micrographs**	19,250	15,978	8,144
**Initial particles**	4,383,273		
**Final particles**	698,565		

Supplementary Table 1. Data collection and image processing information for EhV-ATPase treated with ATP, Mg^{2+}, and Na^{+}.
